# Tobacco promotion 'below-the-line': Exposure among adolescents and young adults in NSW, Australia

**DOI:** 10.1186/1471-2458-12-429

**Published:** 2012-06-12

**Authors:** Donna A Perez, Anne C Grunseit, Chris Rissel, James Kite, Trish Cotter, Sally Dunlop, Adrian Bauman

**Affiliations:** 1 Cancer Institute NSW, Eveleigh, New South Wales, Australia; 2 Sydney School of Public Health, University of Sydney, New South Wales, Australia

## Abstract

### Background

Exposure to tobacco advertising and promotion increases the likelihood of smoking amongst young people. While there is a universal ban on traditional or 'above-the-line' advertising in Australia, the types and extent of exposure of young people to 'below-the-line' tobacco advertising and promotion is largely unknown. In this study we aim to identify levels of exposure of New South Wales (NSW) adolescents and young adults to tobacco promotion at the point-of-sale (PoS), on the internet, in entertainment media and at venues such as events or festivals and pubs, clubs, nightclubs, or bars; and to identify those most at risk of exposure.

### Methods

A telephone survey of 1000 NSW adolescents and young adults aged 12 to 24 years was conducted. Self-reported exposure to tobacco promotions or advertising in the last month were measured in four areas: (1) promotions or advertising at (a) events or festivals and (b) pubs, clubs, nightclubs or bars, (2) on the internet, (3) people smoking cigarettes in (a) movies, (b) TV shows, (c) video games and (d) on the internet, and (4) displays of cigarette packs for sale at (a) large supermarkets, (b) grocery stores or small supermarkets, (c) convenience stores, and (d) service or petrol stations. Smoking status and susceptibility to smoking was also assessed.

### Results

A substantial proportion of the young people surveyed reported seeing tobacco promotion sometimes or often in the last month over most of the channels studied. The highest levels of exposure were at the PoS (approx. two-thirds) and to people smoking cigarettes in movies (77%). Lower levels of exposure to tobacco promotions and imagery were reported on the internet (20%); at events or festivals (22.5%); in pubs, clubs, nightclubs or bars (31%); and in video games (23%). However, the odds of exposure through video games increased by 8% for every additional hour spent on the internet per day.

### Conclusions

This study shows that adolescents and young adults in NSW are exposed to tobacco advertising or promotion at the PoS, on the internet, in entertainment media and at venues such as events or festivals and pubs, clubs, nightclubs or bars, despite the restrictions on the marketing of tobacco in Australia.

## Background

There is considerable evidence linking exposure to tobacco advertising and promotion with an increased likelihood of smoking amongst young people.[1-4] As a result, an increasing number of countries have implemented bans on tobacco advertising, marketing and promotion.[5] Australia's Tobacco Advertising Prohibition Act 1992 and tobacco control legislation in the states and territories has been implemented to prevent most promotion or marketing of tobacco through traditional or 'above-the-line' forms of media (print, radio, television, billboards, and other locations).[6,7]

In New South Wales (NSW), the most populous state of Australia, the Public Health (Tobacco) Act of 2008 introduced new requirements relating to advertising of tobacco products on retail premises, including how tobacco products may be displayed. The new regulations state that tobacco products must be stored out of sight so that they cannot be seen by the public from inside or outside the retail premises. Large retailers (more than 50 employees) went out of sight 1 January 2010 (Phase 1), small retailers (50 or fewer employees) followed from 1 July 2010 (Phase 2) and specialist tobacconists must comply by 1 January 2013 (Phase 3).

Despite the ban on most traditional forms of tobacco advertising and promotion in Australia, the tobacco industry has adapted by diverting resources to non-traditional or 'below-the-line' means of promotion, such as point-of-sale (PoS) displays; portrayal in films or movies; TV programs, magazines and electronic games; internet advertising and events marketing.[7-10]

Large-scale population studies have shown that greater exposure to PoS tobacco displays is associated with an increased likelihood of adolescent smoking.[11-17] A recent study in the United Kingdom (UK), for instance, found that both noticing and being attracted to PoS displays were associated with susceptibility to smoking amongst never smokers aged 11 to 16 years.[18] Furthermore, experimental studies have shown that youth exposed to images of tobacco-saturated PoS displays had stronger perceptions relating to the availability and ease of tobacco purchase and of peer and adult smoking as well as less support for tobacco control policies, compared to youth exposed to images of PoS displays with no tobacco imagery.[19,20]

The appearance of tobacco brands or tobacco related products in cinema films has a long history and is a pervasive form of tobacco promotion.[21] A recent study from the UK showed that tobacco appeared in 70% of the most popular films from 1989 to 2008.[22] A number of studies have shown that greater exposure to smoking in films is associated with an increased likelihood of smoking among adolescents and young adults [23-29], and that this transcends different cultural contexts.[30] Evidence as to the extent and effects of youth exposure to media portrayals of smoking has previously come from countries such as the United States (US) and the UK, however, with little research to date on the level and effect of exposure in Australian youth.

The internet may also influence youth tobacco use because it provides potential access to tobacco products, as well as a venue that may stimulate demand through advertising and promotional messages.[31] In 2005, almost one fifth of adult internet users in the US recalled seeing tobacco products advertised online, with young adults being the most likely group to recall such advertising.[32] Newly emerging forms of marketing include viral marketing through social networking sites in which tobacco company names, logos and images can be prominently displayed and rapidly disseminated amongst users of the sites.[33] This type of marketing, though aimed primarily at young adults, is also likely to influence younger teenagers.[6]

Research investigating online exposure to tobacco promotion has largely focused on adults [32], with little or no research to date focusing on adolescent exposure, despite the wide-spread use of the internet in this age group.[33]

It is important to assess exposure among adolescents and young adults to non-traditional or below-the-line means of tobacco promotion, given this may be undermining tobacco control efforts and diminishing the potential to further prevent uptake or reduce smoking in this group of young people.

This study aims to identify (1) the degree to which NSW adolescents (12-17 years of age) and young adults (18-24 years of age) have been exposed to tobacco promotion at the PoS, on the internet, in entertainment media, and at entertainment venues; and (2) to profile the characteristics of adolescents and young adults most at risk of exposure to these types of tobacco promotion.

## Methods

### Design

The Cancer Institute NSW's Tobacco Promotion Impact Study (TPIS) is a telephone survey of adolescents and young adults aged 12 to 24 years in New South Wales (NSW). It monitors exposure to tobacco promotion at the PoS, on the internet and in entertainment media, and smoking-related cognitions and behaviours. This paper presents findings from the baseline survey conducted in NSW in June 2010.

Households were recruited using random digit dialling (landline telephone numbers only) and participants within households were recruited using random selection (selecting the *n*th oldest eligible person aged 12 to 24 years). Permission was obtained from parents of 12 to 15 year olds before conducting each interview. Participants were interviewed between 8 June and 30 June 2010 (*n *= 1,000). The questionnaire was piloted with the survey population prior to the commencement of fieldwork. An overall response rate of 45% (using the American Association for Public Opinion Research Response Rate #3) was achieved for this period [34], comparable or superior to other similar studies.[35,36] The TPIS was approved by the NSW Population Health Services Research Ethics Committee.

### Measures

#### Individual characteristics

Demographic items capturing age, gender, region (Sydney vs. Rest of NSW), living arrangement, language spoken at home, and disposable income were included in the study. Postcode of residence was coded according to the Socio-Economic Indices for Areas SEIFA; [37], an index of relative disadvantage, to indicate neighbourhood socio-economic status (SES). Quintiles 4-5 were collapsed to indicate low SES, and quintiles 1-3 were collapsed to indicate moderate to high SES. Age was operationalised for the bivariate and multiple variable analyses as a binary variable grouping together participants aged 12-17 years (adolescents), and those aged 18-24 years (young adults).

The amount of time spent on the internet and watching TV was reported as hours and minutes per day and converted to hours per day (maximum of 10 h). Amount of disposable income was measured by asking participants how much money they had available during a normal week to spend on themselves (none, $50 or less, $50-$100, $100-$150, $150-$200, over $200, don't know). The categories were collapsed to none, $50 or less, $50-$100, $100+, unknown for analysis.

#### Smoking status

*Current smokers *were defined as those who had smoked cigarettes in the past month. *Ex-smokers *were those who had ever smoked cigarettes, but not in the past month, and had smoked 100 cigarettes or more in their lifetime. *Experimenters *had ever smoked cigarettes, but not in the past month, and had smoked less than 100 cigarettes in their lifetime. Non-smokers were categorised into one of two groups: *susceptible non-smokers *and *non-susceptible non-smokers*.[38] To determine susceptibility to smoking, participants who had never had a puff of a cigarette were asked "Do you think you will try cigarettes sometime soon", "Do you think you will try cigarettes sometime in the next year" and "If a friend offered you a cigarette, would you try it" (definitely no, probably no, probably yes, definitely yes). *Susceptible non-smokers *were those whose response to any one of the three smoking susceptibility items was anything other than 'definitely no'. *Non-susceptible non-smokers *were those who answered 'definitely no' to all three smoking susceptibility items. For the multiple variable analysis, a binary "ever smoked" variable was used with those who answered "yes" to "have you ever smoked a cigarette, even just a puff" coded as "ever smokers" and those who answered "no" or "don't know" as "never smoked".

#### Smoking exposure

Exposure to smoking in the household and among friends was assessed by recording the number of current smokers in a respondent's household and how many (if any) of the participants' five closest friends smoked. As both of these variables had highly skewed distributions, smoking in the household was operationalised as a three category variable (none, one person, two or more people) and smoking among friends as a four category variable (none, one friend, two friends, three or more friends).

#### Perceived exposure to tobacco promotion

Self-reported exposure to tobacco promotions or advertising in the last month were measured in four areas: (1) promotions or advertising at (a) events or festivals and (b) pubs, clubs, nightclubs or bars, (2) brands, company names or logos on the internet, (3) people smoking cigarettes in (a) movies, (b) TV shows, (c) video games (platform not specified) and (d) on the internet, and (4) displays of cigarette packs for sale at (a) large supermarkets (defined for participants as having more than five cash registers), (b) grocery stores or small supermarkets, (c) convenience stores, and (d) service or petrol stations. Respondents aged 12-15 years (n = 334) were not asked about exposure in pubs, clubs, nightclubs or bars due to the low likelihood they would be attending these venues. Exposure to cigarette pack displays at PoS was measured as, firstly, how often the respondent visited each store type (never, rarely, sometimes, often). Those who had ever visited these stores were then asked how often they had seen displays of cigarette packs for sale in each using the same response frame.

### Statistical analyses

#### Outcome measures on exposure to tobacco promotion

For all tobacco promotion channels except cigarette pack displays, the response categories of never/rarely and sometimes/often were collapsed to create binary exposure variables. Respondents who reported that they didn't know whether they had seen such promotions were coded "never/rarely" for exposure. Those who refused to answer a question were coded as missing for that exposure channel (at maximum n = 2).

Exposure to cigarette pack displays in stores (large supermarkets, small supermarkets, convenience stores and petrol stations) were combined measures reflecting both frequency of visits to the store type and the frequency with which the respondent said they saw cigarette pack displays at that store. For both visit frequency and frequency of seeing pack displays, the coding ranged from 0 (never) to 3 (often). For each store type, the two frequency variables were multiplied to create a combined numeric cigarette pack display exposure score which was subsequently categorised as follows: Low exposure: participants who reported they either never visited that store type or never saw cigarette pack displays (irrespective of store visit frequency) or rarely visited and rarely saw pack displays (score 0 or 1); High exposure: combinations of visit frequency and seeing cigarette pack display frequency of either often and sometimes, or often and often (score 6 or 9); and Medium exposure: all other combinations of store visit and frequency of seeing pack displays (score 2 to 4). For the multiple variable analysis, the outcome variable for exposure to cigarette pack displays in stores was a single binary variable coded high exposure (versus not high) for the exposure score (as calculated above) aggregated over all store types. High exposure was defined as being in the top quartile of the total exposure score.

#### Analytic techniques

Descriptive statistics were generated for the tobacco promotion channels by gender and age group (12-17 and 18-24 years). Bivariate comparisons of level of exposure over all promotion channels across gender and age were examined using chi-square.

Multiple logistic regression models estimated the adjusted odds of participants reporting seeing each of the different types of tobacco promotion sometimes or often. Each model included as predictors: age group (12-17 years, 18-24 years), gender, SES (advantaged, disadvantaged), disposable income, ever smoked, household smokers, and friends smoking. For outcome variables examining tobacco promotion on the internet and in video games, hours per day spent on the internet was also included in the model, and the model examining depiction of people smoking in TV shows also included time spent watching TV (in hours per day). Contrasts with the reference category for multiple category predictor variables were Bonferroni adjusted.

The data were weighted to the NSW population for known age (12-15 years, 16-19 years, 20-24 years), sex (female, male) and region (Sydney, Rest of state) distributions for 12-24 year olds within NSW from the 2006 Australian Bureau of Statistics (ABS) Census data [39] using post-stratification weights. All analyses were conducted using Stata v11.1 [40] and used a threshold of alpha at 0.05 for statistical significance.

## Results

### Description of sample

Table 1 shows the (unweighted) socio-demographic characteristics of the sample of participants from NSW (n = 1000), by age group.

**Table 1:** Socio-demographic characteristics NSW sample (n = 1000) by age group

CHARACTERISTIC	12-17 years n =518		18-24 years n =482		Total
**GENDER**	**n**	**%**	**n**	**%**	**%**
Male	258	49.8	240	49.8	49.8
**REGION**					
Sydney	298	57.5	343	71.2	64.1
Rest of NSW	220	42.5	139	28.8	35.9
**NEIGHBOURHOOD SES**					
Low	163	31.5	148	30.7	31.1
Mod-High	355	68.5	334	69.3	68.9
**LIVING ARRANGEMENT**					
Live with parent(s)/guardians/family	511	98.8	402	83.8	91.6
Live with a, or am sole parent/share with	6	1.2	78	16.25	8.4
others/spouse/live alone					
**LANGUAGE AT HOME**					
English	435	84.0	348	72.2	78.3
**SMOKING STATUS**					
Current smoker	44	8.5	125	25.9	16.9
Non-susceptible non-smoker	322	62.2	155	32.2	47.7
Susceptible non-smoker	84	16.2	23	4.8	10.7
Ex-smoker	1	0.2	23	4.8	2.4
Experimenter	67	12.9	156	32.4	22.3
**FRIENDS SMOKING†**					
None	331	63.9	149	30.9	48.0
1 friend	73	14.1	101	21.0	17.4
2 friends	55	10.6	81	16.8	13.6
3+ friends	59	11.4	151	31.3	21.0
**HOUSEHOLD SMOKING**					
No one	380	73.4	285	59.1	66.5
1 person	96	18.5	133	27.6	22.9
2+ people	42	8.1	64	13.3	10.6
**DISPOSABLE INCOME**					
None	51	9.9	9	1.9	6
<=$50	332	64.1	76	15.8	40.8
$50-$100	68	13.1	70	14.5	13.8
$100+	48	9.3	310	64.3	35.8
Unknown	19	3. 7	17	3.5	3.6
INTERNET USE* (n = 991)	**median**	**mean**	**median**	**mean**	**Mean**
(minutes per day)	120	120.5	120	171.8	148.1
TV USE*					
(minutes per day)	90	108.8	90	103.6	106.0
TV USE*					
(minutes per day)	90	108.8	90	103.6	106.0

* Internet and TV mean use in minutes weighted to state population by age and gender.Values exceeding 600 min (10 h, <1% of sample) recoded to 600.

† How many of five closest friends smoke.

Just under half the sample were male and were aged 18-24 years. Almost one-third lived in areas categorised as disadvantaged by SEIFA index. The majority of participants lived with their parents or guardians, although one-sixth of young adults (18-24 years) lived away from their parents. Most participants were not currently smoking (83%), and reported that no one in their household (67%) smoked, however just over half had friends who smoked. Just under two-thirds of participants aged 12-17 years had less than $50 in disposable income per week, while the same proportion of young adults had $100 or more for discretionary spending. Median internet use and time spent watching TV was two hours and 1.5 h per day respectively and was the same for both the adolescents and young adults.

### Bivariate analyses of tobacco promotion

The proportion of participants reporting having seen tobacco promotion in the past month and results of the bivariate comparisons of tobacco promotion exposure across age group and gender are shown in Table 2.

**Table 2:** Proportions in NSW exposed to forms of tobacco promotion by gender and age (n = 1000)

EXPOSURE	Male %	Female %	*p*	12-18 years %	18-24 years %	Total *p*
**PROMOTIONS OR ADVERTISING**						
**Events/festivals**			NS			=.001
Never/rarely	79.7	75.2		72.8	81.6	77.5
Sometimes/often	20.3	27.8		27.2	18.4	22.5
Pubs/clubs/nightclubs/bars*			NS			=.033
Never/rarely	71.4	67.2		75.9	67.3	69.2
Sometimes/often	28.9	32.8		24.1	33.7	30.8
**BRANDS, COMPANY NAMES OR LOGOS**						
**Internet**			NS			<.001
Never/rarely	81.9	78.2		73.9	85.5	80.1
Sometimes/often	18.1	21.8		26.1	14.5	19.9
**PEOPLE SMOKING CIGARETTES**						
**Movies**			NS			NS
Never/rarely	24.1	21.5		22.6	23.0	22.8
Sometimes/often	75.9	78.5		77.4	77.0	77.2
**TV shows**			NS			NS
Never/rarely	34.6	29.3		29.2	34.4	32.0
Sometimes/often	65.4	70.7		70.8	65.6	68.0
**Video games**			<.001			NS
Never/rarely	66.7	87.2		76.5	77.0	76.8
Sometimes/often	33.3	12.8		23.6	23.0	23.3
**On the internet**			NS			NS
Never/rarely	75.3	73.5		74.1	74.7	74.4
Sometimes/often	24.7	26.5		25.9	25.3	25.6
**CIGARETTE PACK DISPLAYS†**						
**Large supermarkets**			=.018			<.001
Low	35.6	30.2		23.9	40.8	32.9
Med	33.5	30.3		36.8	27.8	31.9
High	30.9	39.5		39.3	31.5	35.1
**Small supermarkets**			<.001			<.001
Low	39.2	28.1		25.6	40.8	33.7
Med	36.4	32.4		37.7	31.6	34.4
High	24.4	39.6		36.7	27.6	31.8
**Convenience stores**			=.001			NS
Low	41.1	31.7		34.5	38.2	36.5
Med	38.7	39.4		40.9	37.4	39.0
High	20.3	28.9		24.6	24.4	24.5
**Petrol stations**			NS			=.014
Low	30.4	30.3		28.0	32.4	30.4
Med	33.7	32.9		38.1	29.2	33.3
High	35.9	36.8		34.0	38.4	36.3

† Level of exposure determined by combination of frequency visiting site and frequency seeing pack displays.

* Respondents aged 12-15 years excluded (n = 334) as not asked this question - younger age group 16-17 year-olds only.

Overall, a substantial proportion of the young people surveyed reported seeing tobacco promotion sometimes or often in the last month over most of the channels. The highest rate was for seeing people smoking in movies (77%) and the lowest was for seeing tobacco brands, company names or logos on the internet (20%). Around one-third each reported seeing cigarette pack displays in low, medium, or high frequency and this distribution was similar across the different store types (large and small supermarkets, convenience stores and petrol stations).

Bivariate analyses of reported tobacco promotion exposure for the different channels by gender showed few differences except more male participants saw people smoking sometimes/often in video games (33% vs. 13%, *p *< 0.001) than females, while female participants reported seeing cigarette pack displays at high frequency in large supermarkets, small supermarkets and convenience stores more often than males.

Comparisons of exposure to tobacco promotion by age showed adolescents were more likely than the young adults to report seeing brands, company names or logos on the internet, promotions/advertising at events/festivals, and cigarette pack displays in large and small supermarkets, but had lower exposure to promotions/advertising at pubs/clubs/nightclubs/bars (16-17 year olds only). Young adults reported higher levels of exposure to cigarette pack displays in petrol stations. No differences by age were observed for seeing people smoking cigarettes in movies, on TV shows, in video games or on the internet.

### Multiple variable analyses of tobacco promotion

Table 3 shows results of the significant adjusted models of exposure to tobacco promotion at events/festivals, on the internet, in video games and cigarette pack displays in all stores combined.

**Table 3:** Adjusted odds ratios with 95% percent confidence intervals^1 ^for participants reporting seeing forms of tobacco promotion sometimes/often in the last month

	Events/festivals			Brands/companynames/logos on internet			People smoking in video games			Cigarette pack displays exposure all stores		
Predictor (reference category)	**OR**	**95%CI**	**p**^3^	**OR**	**95%CI**	**p**^3^	**OR**	**95%CI**	**p**^3^	**OR**	**95%CI**	**p**^2^
**Age 18-24 years (12-17)**	0.68	(0.46,1.01)	0.055	**0.46**	**(0.30,0.70)**	**0.000**	0.90	(0.59,1.38)	0.626	**0.61**	**(0.40,0.91)**	**0.016**
**Sex (Male)**	1.32	(0.97,1.80)	0.078	1.23	(0.88,1.70)	0.219	**0.28**	**(0.20,0.39)**	**<.001**	**1.58**	**(1.17,2.13)**	**0.003**
**SES (not disadvantaged)**	0.95	(0.68,1.32)	0.748	1.31	(0.92,1.85)	0.134	1.01	(0.71,1.44)	0.943	1.13	(0.82,1.56)	0.437
**Disposable income (none)**			0.354			0.298			0.976			0.947
<=$50	1.52	(0.79,2.91)	0.825	1.90	(0.87,4.11)	0.420	0.82	(0.43,1.56)	1.00	0.98	(0.5,1.90)	1.00
$50-$100	1.10	(0.52,2.33)	1.00	2.34	(1.01,5.42)	0.188	0.80	(0.38,1.68)	1.00	1.64	(0.78,3.45)	1.00
$100+	1.05	(0.50,2.20)	1.00	1.72	(0.73,4.03)	0.848	0.80	(0.39,1.64)	1.00	1.60	(0.75,3.40)	1.00
Unknown	0.94	(0.32,2.75)	1.00	1.26	(0.37,4.32)	1.00	0.75	(0.23,2.46)	1.00	1.06	(0.38,2.99)	1.00
**Ever smoked (never smoked)**	0.85	(0.58,1.27)	0.435	**0.64**	**(0.43,0.95)**	**0.027**	0.83	(0.55,1.23)	0.345	1.27	(0.86,1.87)	0.232
**Household smoking (none)**			0.457			0.701			0.646			0.303
1 person	1.26	(0.86,1.85)	0.461	1.18	(0.8,1.75)	0.818	0.84	(0.55,1.26)	0.775	0.86	(0.59,1.25)	0.474
2+ people	0.99	(0.57,1.75)	1.00	1.01	(0.55,1.85)	1.00	0.85	(0.50,1.45)	1.00	0.66	(0.38,1.15)	1.00
**Friends smoking (none)**			**0.015**			0.079			0.055			0.161
1 friend	**1.98**	**(1.28,3.04)**	**0.006**	1.70	(1.09,2.66)	0.059	1.18	(0.74,1.88)	1.00	1.29	(0.85,1.97)	0.403
2 friends	1.04	(0.63,1.74)	1.00	1.50	(0.91,2.48)	0.340	1.90	(1.16,3.11)	0.031	1.26	(0.79,2.01)	0.459
3+ friends	1.28	(0.79,2.08)	0.953	1.52	(0.92,2.49)	0.302	1.58	(0.98,2.55)	0.187	0.95	(0.59,1.52)	0.101
**Internet use (hours/day)**		-	-	1.06	(0.99,1.15)	0.11	**1.08**	**(1.01,1.17)**	**0.030**	-	-	-

^1 ^Full results shown only for models that reached statistical significance. Analyses also included seeing promotions or advertisements in pubs/clubs/nightclubs or bars, seeing people smoking in movies, in TV shows and on the internet, but the overall models did not reach statistical significance (>.05)

**^2 ^**High exposure for seeing cigarette pack displays defined as top quartile of scores for summed frequency of visit by frequency of seeing cigarette pack display across all store types

^3 ^Significance values for overall test shown for multicategory variables (e.g., disposable income), and multiple contrasts reported with Bonferroni adjustment

Overall models for exposure to promotion/advertising in pubs/clubs/nightclubs/bars, seeing people smoking cigarettes in movies, in TV shows or on the internet did not reach statistical significance and therefore are not further described.

For tobacco promotion/advertising at events/festivals, only the number of friends who smoked was significantly related to seeing this type of promotion once adjusted for all other variables. Those with one smoking friend compared with no friends who smoked had almost twice the odds of reporting seeing this type of promotion sometimes or often.

The odds of seeing cigarette brands, tobacco company names or logos on the internet were more than halved among participants aged 18-24 years compared with adolescents, and those who had ever smoked had odds of reporting seeing this type of promotion around one-third lower than those who had never smoked.

The odds of reporting seeing people smoking cigarettes in video games sometimes or often were over 70% lower for female participants. Odds of exposure through video games also increased by 8% for every additional hour spent on the internet per day.

High exposure to cigarette pack displays was more likely for female compared with male participants (Adjusted Odds Ratio (AOR) =1.58, *p *= .003), but less likely for participants aged 18-24 years than those aged 12-17 years (AOR = .61, *p *= .016).

## Discussion

This study shows that adolescents and young adults in NSW are exposed to relatively high levels of tobacco advertising or promotion at the PoS, on the internet, in entertainment media and at venues such as events or festivals and pubs, clubs, nightclubs or bars, despite the restrictions on the marketing of tobacco in Australia. The high levels of exposure particularly at the PoS and to people smoking cigarettes in movies and on TV found in this study highlight that the retail environment and entertainment media are important avenues for the industry to market tobacco and tobacco products.

Most adolescents and young adults were exposed to cigarette pack displays in stores in NSW, which provides further justification for the introduction of the PoS display ban. It is interesting to note, however, that despite the first phase of the PoS display ban in NSW being implemented six months prior, most adolescents and young adults still report being exposed to cigarette pack displays in these stores. This raises the question of whether participants actually saw the displays, indicating non-compliance with legislation, or if they assumed that they had seen them because they had grown used to seeing the displays prior to Phase 1.

Perceived exposure to people smoking cigarettes in movies and on TV was also found to be relatively high in this study and efforts to reduce exposure warrants attention in Australia. Although no association was found between exposure and smoking, other studies have shown how portrayals of smoking in films or movies can increase susceptibility to smoking [23].

Exposure to people smoking cigarettes in video games; tobacco brands, company names and logos on the internet; and promotions or advertising for cigarettes or other tobacco products at events or festivals, or in pubs, clubs, nightclubs or bars, while comparatively low, was still reasonably common. In particular, almost one-quarter of participants were using the internet for three or more hours each day, making the potential for exposure via this medium quite high. Whilst no studies to the authors' knowledge have assessed the impact of these forms of exposure on smoking-related cognitions and behaviours among adolescents and young adults, it is possible that exposure to smoking through these mediums may be undermining efforts to denormalise smoking.

This study demonstrates that adolescents in particular were at highest risk of exposure to 'below-the-line' means of tobacco advertising or promotion, and that tobacco industry marketing through the mediums studied are reaching this vulnerable population.

### Strengths and limitations

Strengths of the TPIS are the large sample size, the representativeness of the sample, the inclusion of a large number socio-demographic measures and that fact that it is the only known study of its type in Australia monitoring exposure to tobacco promotion in 'below-the-line' channels (at the PoS, on the internet and in entertainment media) among adolescents and young adults.

There are, however, some limitations. Firstly, the study relies on self-reported exposure not actual exposure to tobacco promotion. The measure of exposure to tobacco promotion across a variety of channels relied on retrospective recall, possibly resulting in some imprecision in measurement. Similarly, while the distinction between small and large chain grocery stores in the Australian context is generally very obvious, this may also have resulted in some imprecision in measurement. Secondly, the use of RDD sampling frame will have resulted in systematic exclusion of mobile phone-only households. However, this should not have overtly influenced the results as the proportion of mobile-phone only households in Australia is relatively small [41,42]. Finally, the results from this study may not be generalisable to adolescents and young adults from other jurisdictions given the potential differences in tobacco control legislation regarding promotion or marketing of tobacco.

## Conclusions

Findings from this study highlight that 'below-the-line' means of tobacco promotion are being noticed by young people in NSW, with high levels of exposure at the PoS through cigarette pack displays and to portrayals of smoking in films or movies and TV shows.

Exposure to tobacco promotion through video games, the internet and events is also evident and of particular concern are the higher levels of exposure among adolescents aged 12-17 years. Public health authorities need to not only monitor these trends, but to address tobacco promotion in these forms through policy and practice.

## Competing interests

The authors declare that they have no competing interests.

## Authors' contributions

DAP conceived of the study, participated in its design and coordination, oversaw the statistical analysis and drafted the manuscript. ACG conceived and performed the statistical analysis, led the interpretation of results and helped to draft the manuscript. CR advised on the statistical analysis, aided in the interpretation of results and helped to draft the manuscript. JK participated in the design of the study and helped to draft the manuscript. TC participated in the design of the study and helped to draft the manuscript. SD participated in the design of the study, advised on the statistical analysis and critically reviewed the manuscript. AB advised on the statistical analysis, aided in the interpretation of results and critically reviewed the manuscript. All authors read and approved the final manuscript.

## Pre-publication history

The pre-publication history for this paper can be accessed here:

http://www.biomedcentral.com/1471-2458/12/429/prepub
